# Photoactive Tweezers

**DOI:** 10.1002/adma.74255

**Published:** 2026-07-23

**Authors:** Jingyuan Chen, Qingxin Guo, Shiwei Zhang, Donghao Cui, Changjin Wu, Pei Xu, Wei Wang, Kai Lou, Binglin Zeng, Jinyao Tang

**Affiliations:** ^1^ Department of Chemistry The University of Hong Kong Hong Kong China; ^2^ Materials Innovation Institute for Life Sciences and Energy (MILES) HKU‐SIRI Shenzhen China; ^3^ Department of Materials Science and Engineering Harbin Institute of Technology Shenzhen Guangdong China; ^4^ Shenzhen Kayja‐Optics Technology Co. Ltd. Shenzhen Guangdong China; ^5^ HKU‐CAS Joint Laboratory on New Materials and Department of Chemistry The University of Hong Kong HongKong China

**Keywords:** micromanipulation, photoactive colloids, self‐trapping

## Abstract

The manipulation of micro/nanoscale objects plays a critical role across a wide range of scientific and engineering disciplines. Photoactive colloids, capable of converting low‐intensity light into autonomous motion, present a highly programmable platform for micromanipulation. In this work, we introduce a “photoactive tweezers” system based on visible‐light‐driven, dye‐sensitized TiO_2_ particles. These particles exhibit positive phototaxis and are autonomously confined within structured optical fields via a self‐electrophoretic mechanism. Combining experiments and simulations, we reveal that a circular optical trap creates an effective harmonic potential well with tunable stiffness, controlled by the ratio of the particle size to the optical spot size. Using dynamic light patterns, we further showcase the platform's versatility through shape‐tunable confinement, wavelength‐selective trapping, and reconfigurable assembly of colloidal lattices. Moreover, we demonstrate that individual or collective active colloids can function as autonomous manipulators, transporting cargo larger than themselves and driving collaboratively assembled micromachines. This approach establishes a robust, flexible, and accessible strategy for advanced micromanipulation and active matter research.

## Graphical abstract

A photoactive tweezers system based on photoactive colloids is developed and systematically investigated in this study. Through experimental and theoretical methods, we demonstrate that the photoredox reaction catalyzed by a dye‐sensitized TiO_2_ particle generates a strong positive phototaxis, enabling self‐entrapment in the beam center. This work presents an effective strategy for controlling the confined motion of photoactive colloids as well as the transportation of cargoes through a real‐time, spatially modulated light field.

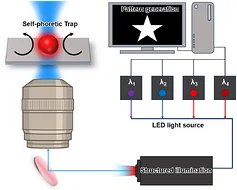

## 1 Introduction

The precise manipulation of micro/nanoparticles is pivotal to modern microscale science and technology, with wide‐ranging applications in biophysics, materials assembly, and lab‐on‐a‐chip systems [1, 2, 3]. Among the diverse techniques developed, including optical [4, 5, 6], magnetic [7, 8, 9], and optoelectronic tweezers [10, 11, 12], light‐based approaches are particularly attractive due to their non‐contact operation and high degree of programmability. However, conventional optical trapping techniques typically demand high‐intensity laser beams to generate sufficient trapping forces, whereas alternative strategies based on photothermal or electric field effects often rely on pre‐patterned substrates [4, 13, 14]. An ideal platform that combines optical programmability with low‐power operation and substrate independence remains highly desirable.

Photoactive colloids, which can convert light energy into autonomous motion via surface photochemical reactions, offer a novel pathway to address this challenge [15, 16]. Upon illumination, these particles generate localized chemical concentration gradients, leading to an electric potential that induces diffusiophoresis or electrophoresis, thereby propelling them through the surrounding medium [17, 18, 19, 20]. This self‐propulsion can be exquisitely controlled by light, providing inherent wavelength selectivity and dynamic programmability [21, 22]. Previous studies have demonstrated that structured illumination can guide individual active particles [23, 24, 25] and trigger collective behavior in ensembles [21, 23, 24, 26, 27, 28]. Such intrinsic motility and optical steerability make these particles not only a form of active matter but also potential candidates for building programmable manipulation tools. However, the concept of leveraging these particles, particularly their ability to be controlled by light‐induced phoretic forces, to construct a self‐contained tweezer system capable of trapping and manipulating other passive objects has not been explored.

Here, we realize this concept by introducing “photoactive tweezers” based on dye‐sensitized TiO_2_ colloids. We demonstrate that under structured light spots, these particles undergo phototactic self‐trapping via a light‐induced phoretic mechanism. We quantitatively characterize the trapping potential and corroborate it with simulations. The platform's programmability is demonstrated by dynamically reshaping particle assemblies and achieving reversible transformations between multiple distinct lattice configurations. Furthermore, by harnessing the hydrodynamic flows generated by the active colloids, we extend the functionality to propelling inert cargo several times larger than the particles themselves and coordinating collective micromachines. Unlike conventional tweezers that directly exert forces on passive targets, this work establishes an indirect, actuator‐based manipulation paradigm. Here, light programs and traps active particles, which in turn act as programmable “microtools” to manipulate passive objects via hydrodynamic interactions. This work establishes a versatile micromanipulation platform that operates with standard LED illumination and on conventional substrates, significantly broadening the scope of accessible optical manipulation technologies.

## 2 Results

### 2.1 Demonstration of Phototactic Self‐Trapping

To experimentally investigate the self‐trapping phenomenon of active particles via light‐mediated positive phototaxis, we designed a model system based on photocatalytic semiconductor microparticles. We synthesized monodisperse spherical TiO_2_ particles with a diameter of 2.5 µm and functionalized them with thesensitizer dye LEG4 to extend their light absorption into the visible spectrum (Figure S1). The resulting red‐colored particles were dispersed in a 0.1 M solution of ferrocene (acting as an electron donor) in acetonitrile [29, 30]. This suspension was then sealed within a rectangular glass capillary using wax, which served as the experimental chamber for microscopic observation.

**FIGURE 1:**
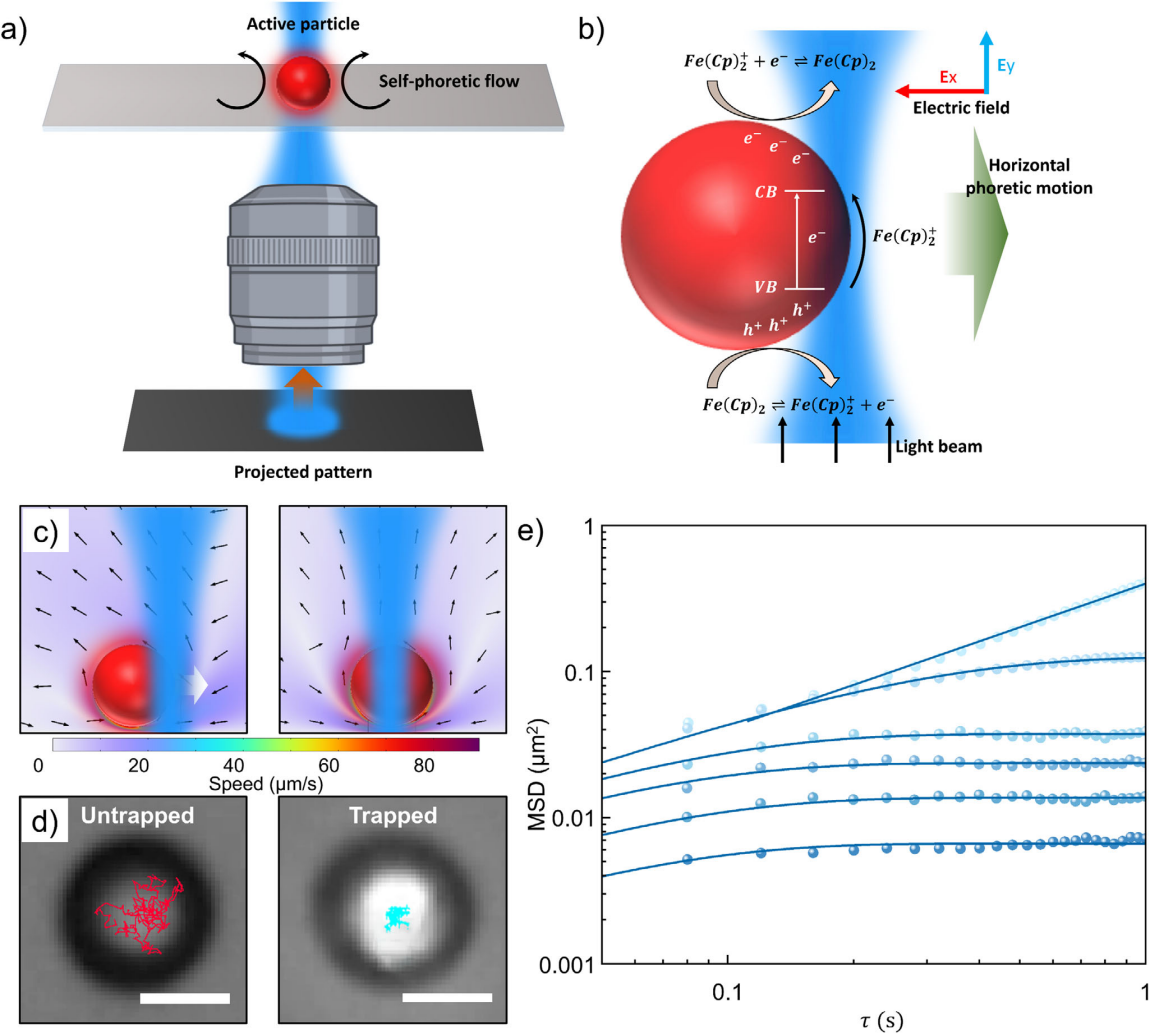
Working principle and characterization of the photoactive tweezers system. (a) Schematic of the experimental setup. Structured blue‐light patterns are projected through a 100× objective of an inverted microscope, generating microscopic optical traps that confine the photoactive particles. (b) Mechanism of positive phototaxis via self‐electrophoresis. The particle moves toward the light source due to a self‐generated, light‐induced electroosmotic flow. (c) COMSOL simulation of the self‐electrophoretic flow around a particle under asymmetric illumination. Left panel: With the particle offset from the beam center, an asymmetric flow field generates a net force that drives it toward the intensity maximum. Right panel: When centered, the flow field is symmetric, resulting in a stable equilibrium. (d) Representative trajectories of LEG4‐TiO_2_ particles in free motion (left) and when optically confined (right). (e) Mean squared displacement (MSD) of a trapped particle as a function of time delay τ for different trapping light intensities. Curves from top to bottom correspond to light intensities of 0, 208, 622, 1037, 1452, and 1866 mW·cm^−2^, respectively. The solid lines are exponential fits of the form *y* = *a*(1 − *e* ^−*bx*^). The different plateau levels indicate progressively stronger confinement at higher light intensities. Scale bars: (d) 2 µm.

As shown in Figure 1a, a customized optical setup was employed. A 465 nm blue light pattern was projected through a structured light device (Four‐Channel Digital Light Processing Projection System, Kayja‐Optics) attached to an inverted microscope. This system allowed for precise regulation of illumination conditions, including the size, shape, number, and real‐time positions of the light spots, along with the light intensity and wavelength.

Under blue illumination, the LEG4‐TiO_2_ particles exhibit positive phototactic behavior that is attributed to a self‐electrophoresis mechanism [31, 32]. The underlying photophysics involves several key steps: photons absorbed by the LEG4‐sensitizer induce charge separation, generating electron‐hole pairs within the TiO_2_ particle. This drives the oxidation of ferrocene (Fe(Cp)_2_) to ferrocenium (Fe(Cp)_2_^+^) on the illuminated hemisphere. Simultaneously, the complementary reduction of ferrocenium ions back to ferrocene occurs on the shaded side. This asymmetric redox reaction establishes a local electric field across the particle, directed from the illuminated to the dark side [33]. Since the particles possess a negative surface charge in the solution, the resulting electrophoretic force propels them directly toward the light source. Notably, when illuminated from below by an off‐center light spot, the particle experiences a horizontal component of the electric field (**E**_x_), resulting in directed movement toward the light spot center (Figure 1b).

To gain deeper insight into the hydrodynamics of this phototactic process, we performed numerical simulations of the self‐electrophoretic flow around an active particle under biased illumination using COMSOL Multiphysics. As displayed in Figure 1c and Figure S2, the off‐center illumination generates a pronounced asymmetry in the slip flow field around the particle. This asymmetric flow pushes the particle toward the beam center. Once the particle center aligns with the spot center, the phoretic flow field becomes symmetric, resulting in stable trapping with no net propulsive force. This self‐induced restoration to a symmetric state effectively confines the particle's movement within the spot, functioning as a highly effective “photoactive tweezers” system without the need for high‐power lasers or complex substrate preparation. We term this system “photoactive tweezers” because the light pattern stably confines the active particle itself within a harmonic potential well, analogous to an optical trap.

Direct experimental evidence of trapping is presented in Figure 1d, which compares the trajectories of a freely diffusing particle and a particle confined within a 1.5 µm diameter light spot. The trajectory of the trapped particle is markedly constrained, exhibiting a substantial reduction in its vibration amplitude compared to the free particle (Movie S1). As shown in Figure 1e, for a fixed light spot size of 1.5 µm, the mean square displacement (MSD) as a function of time delay τ grows significantly more slowly under higher light intensities, and the MSD plateau value decreases monotonically with increasing light intensity. This progressive suppression of the MSD indicates increasingly stronger confinement of the particle's motion within the light spot, consistent with the enhancement of the phototactic restoring force at higher illumination levels. Compared to the freely diffusing LEG4‐TiO_2_ particle (MSD  =  4*D*_0_τ, with *D*_0_  = 0.4 µm^2^ · s^−1^), the trapped particles exhibit a clear transition from free diffusion to confined motion, providing compelling qualitative evidence for effective phototactic self‐trapping.

### 2.2 Theoretical Analysis

**FIGURE 2:**
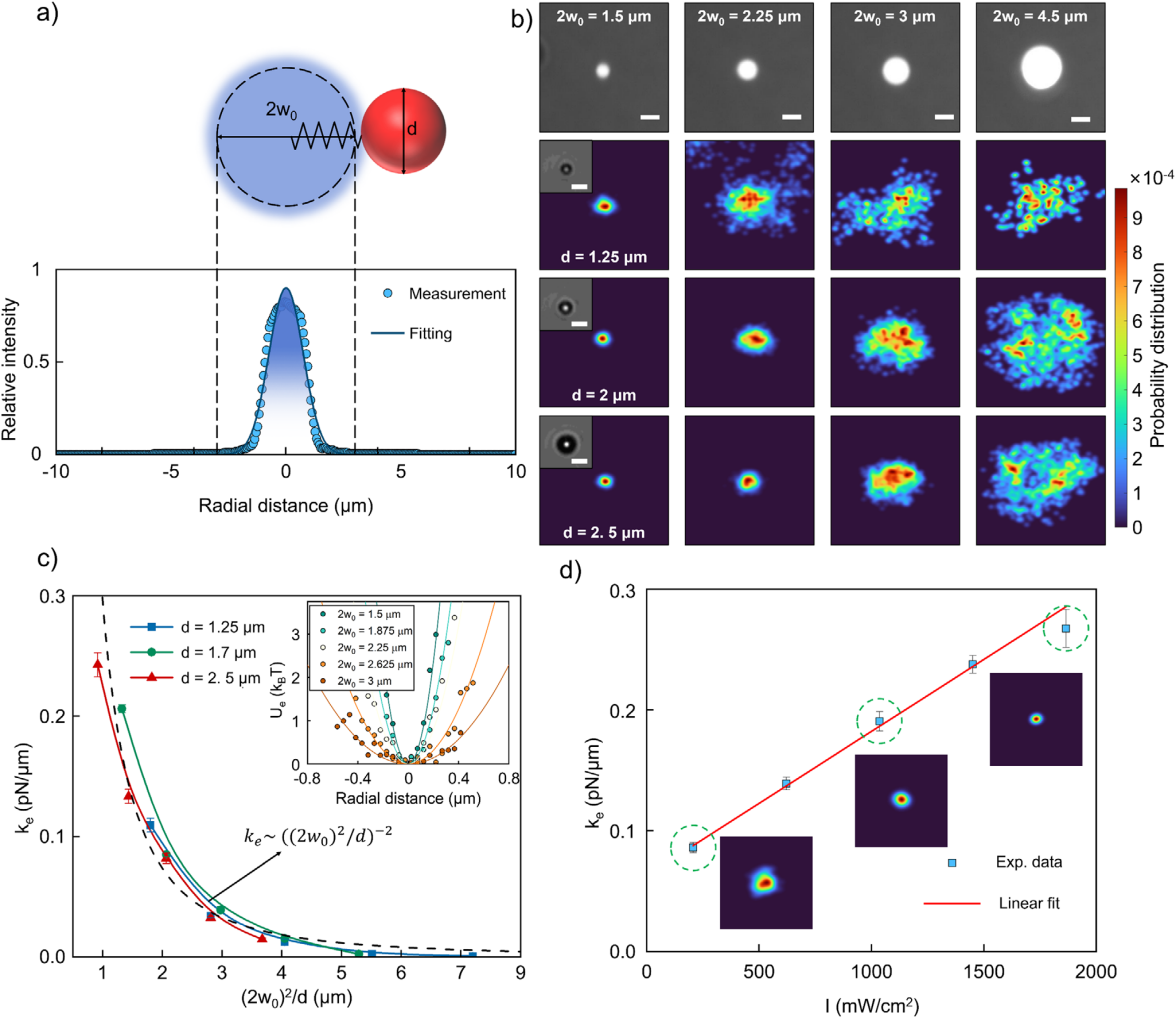
Single‐particle confinement in a circular optical trap. (a) Characterization of the optical trap. A circular light spot is approximated as a Gaussian beam with waist 2*w*_0_. The upper model illustrates the relative scale of the particle diameter (*d*) to the optical beam waist (*w*_0_), while the lower panel shows the light intensity profile. Dots: measured transverse intensity profile. Dark blue line: Gaussian fit. (b) Probability density distributions (main panels) of a single LEG4‐TiO_2_ particle for different combinations of beam waist 2*w*_0_ = 1.5, 2.25, 3, and 4.5 µm and particle diameter *d* = 1.25, 2, and 2.5 µm. Corresponding microscope images of the light spots (top row) and of the particles (left column, insets) are shown. (c) Effective trap stiffness *k_e_* as a function of the relative beam size (2*w*_0_)^2^/*d*. Solid line: fit to $k_{e}∼{\left({\left(2w_{0}\right)}^{2}/d\;\right)}^{-2}$ with *R* ^2^ = 0.919. Inset: Effective harmonic trapping potential (symbols) derived from the probability distribution in (b) for *d* = 2.5 µm, with a parabolic fit (line). (d) Effective trap stiffness *k_e_* as a function of light intensity *I*. Solid line: linear fit ($k_{e}∼I$) with *R* ^2^ = 0.996. Inset: Probability distribution colormaps (same color bar as in (b)) for the intensity conditions marked by green circles. (c, d) Error bars represent the fitting error of *k_e_*. Scale bars: (b) 2 µm.

To better understand the trapping behavior of active colloids within the light spot, we propose a simple spring‐like model analogous to that used in traditional optical tweezers. As sketched in Figure 2a, the light intensity profile is measured and approximated by a Gaussian distribution (lower panel), where a beam waist *w*_0_ can be extracted. The attractive force exerted on the particle by the circular light spot is modeled as a linear restoring force analogous to Hookean spring dynamics. Consistent with optical tweezer systems, a key parameter characterizing the trapping potential is the stiffness of the photoreaction‐induced potential well, which depends not only on the light intensity and beam waist, but also on the particle diameter. Prior to theoretical analysis, we systematically investigated the trapping dynamics of LEG4‐TiO_2_ particles with varying diameters under different beam waists. The spatial probability distribution of each particle over a 40‐second trajectory was computed using the Kernel Density Estimation (KDE) method. The resulting heatmaps (Figure 2b) show that the particle's spatial distribution broadens with increasing beam waist or decreasing particle diameter, indicating weaker confinement (Movie S2).

To quantify the trapping strength, we extracted the effective trap stiffness from the steady‐state probability distribution function *f*(*x*, *y*). Although the system operates out of equilibrium, the particle distribution attains a dynamic steady state within the observation period of 40 s, governed by the balance between the active restoring force and thermal diffusion. To elaborate, when a particle deviates from the center of the light spot, the direction of its self‐electrophoretic force always points toward the center of the spot, acting as a restoring force. When the particle is at the center of the spot, the net driving force is zero; the particle does not undergo net active propulsion, and its instantaneous motion is dominated by Brownian diffusion. This steady‐state distribution allows the definition of an effective potential, which is equivalent to performing a Boltzmann inversion *U_e_* (*x*,*y*) =   − *k_B_T*ln (*f*(*x*,*y*))  [34], where *k_B_* is the Boltzmann constant and *T* is the absolute temperature. Subsequently, the coordinates of the particle were converted into radial distances from the center of the light spot, allowing us to determine the relationship between the trapping potential energy *U_e_* (*r*) and the radial distance *r*. As shown in the inset of Figure 2c, for all tested light spot sizes, *U_e_* (*r*) significantly increases with increasing radial distance *r*. By fitting the experimental data to the harmonic potential form $U_{e}=\frac{1}{2}k_{e}r^{2}$ based on Hooke's law, the effective trap stiffness *k_e_* was extracted. We find that the measured stiffness for different particle diameters and beam waists follows an empirical scaling relation $k_{e}∼{\left({\left(2w_{0}\right)}^{2}/d\right)}^{-2}$ (Figure 2c), and exhibits a linear dependence on the incident light intensity, $k_{e}∼I$ (Figure 2d and Movie S3).

The observed scaling of the effective trap stiffness $k_{e}∼d^{2}/w_{0}^{4}$ originates from the coupling between the Gaussian light profile and the particle's self‐electrophoretic motion mechanism [31, 35]. Note that the particle's phoretic velocity *v_p_* arises from a surface slip flow *v_s_* driven by light induced ionic concentration gradients. For a photoactive particle moving inside a Gaussian beam, the light intensity profile $I\left(r\right)=I_{0}\exp\left(\;-\frac{2r^{2}}{w_{0}^{2}}\right)$ establishes a nonuniform flux distribution, where $I_{0}=\frac{2P}{\pi w_{0}^{2}}$, *P* is the input power from the light source.

The resulting concentration gradient, and thus the phoretic velocity is driven by the intensity gradient, ∇*I*. Near the beam center (*r* ≪ *w*_0_), the magnitude of this gradient approximately scales as $\nabla I\propto \frac{I_{0}r}{w_{0}^{2}}=\frac{2Pr}{\pi w_{0}^{4}}$. Since the self‐electrophoretic velocity is proportional to both the particle diameter *d* (Figure S3) and the light intensity gradient (proportional to concentration gradient), we have *v_p_* ∝*d*∇*I*. In the overdamped regime, this propulsion force is balanced by the Stokes drag force, *F_drag_* = 3πη*dv_p_*, leading to a net restoring force $F=-k_{e}r\propto \frac{d^{2}}{w_{0}^{4}}r$ that confines the particle. The effective trap stiffness is therefore derived as:

$$k_{e}=\frac{dF}{dr}\propto \frac{d^{2}}{w_{0}^{4}}$$

This expression accounts for the observed scaling law. A full derivation process is provided in the Supporting Information. The *d* ^2^ dependence results from the particle's diameter influencing both the slip velocity and the drag force. The strong $w_{0}^{-4}$ dependence arises from two factors: first, the peak intensity *I*_0_ scales as $P/w_{0}^{2}$, and second, the intensity gradient |∇*I*| introduces an additional $1/w_{0}^{2}$ dependence, leading to an overall $1/w_{0}^{4}$ scaling. It is worth noting that several factors limit the applicability of this scaling law. First, it assumes an ideal Gaussian beam profile. The law is not expected to hold quantitatively for structured light patterns with non‐Gaussian intensity distributions. Second, the model assumes a spherical particle shape, where self‐propulsion vanishes at the trap center. For particles with significant shape asymmetry, a persistent net propulsion may arise, altering the confinement dynamics and invalidating the scaling. Nevertheless, our experiments are conducted within the regime where these assumptions are valid, ensuring the consistency between the model and observations.

**FIGURE 3:**
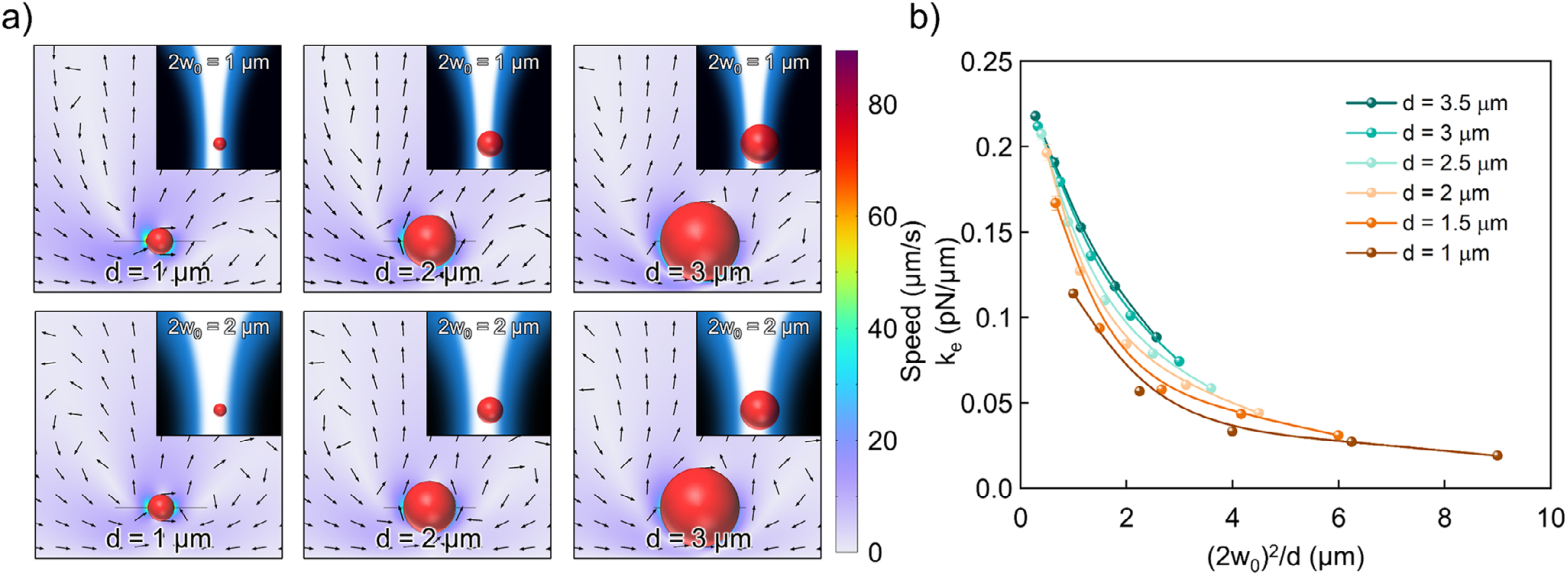
COMSOL simulation of the trapping behavior. (a) Simulated flow field (black arrows) around an active particle under Gaussian beam illumination (inset), with a lateral offset of 0.8 µm between the particle and beam centers. (b) Effective trap stiffness *k_e_* calculated from the simulated, position‐dependent horizontal restoring force generated by the self‐electrophoretic flow.

To further validate the trapping mechanism, we performed COMSOL simulations of the self‐electrophoretic flow. A 2D finite element model was developed, incorporating reactive species diffusion, electrostatic fields, and electroosmotic flow. The light induced reactive flux at the particle surface was defined according to its position relative to the Gaussian beam profile. Figure 3a illustrates the simulated flow fields around particles of different diameters under varying beam waists. When the particle is systematically displaced from the beam center, the resulting net horizontal force increases linearly with displacement, confirming a spring‐like restoring force. By simulating a range of particle diameters and beam waists, we successfully reproduced the experimental scaling of effective trap stiffness (Figure 3b), in good agreement with the measurements presented in Figure 2c.

### 2.3 Versatile Trapping via Structured Light

**FIGURE 4:**
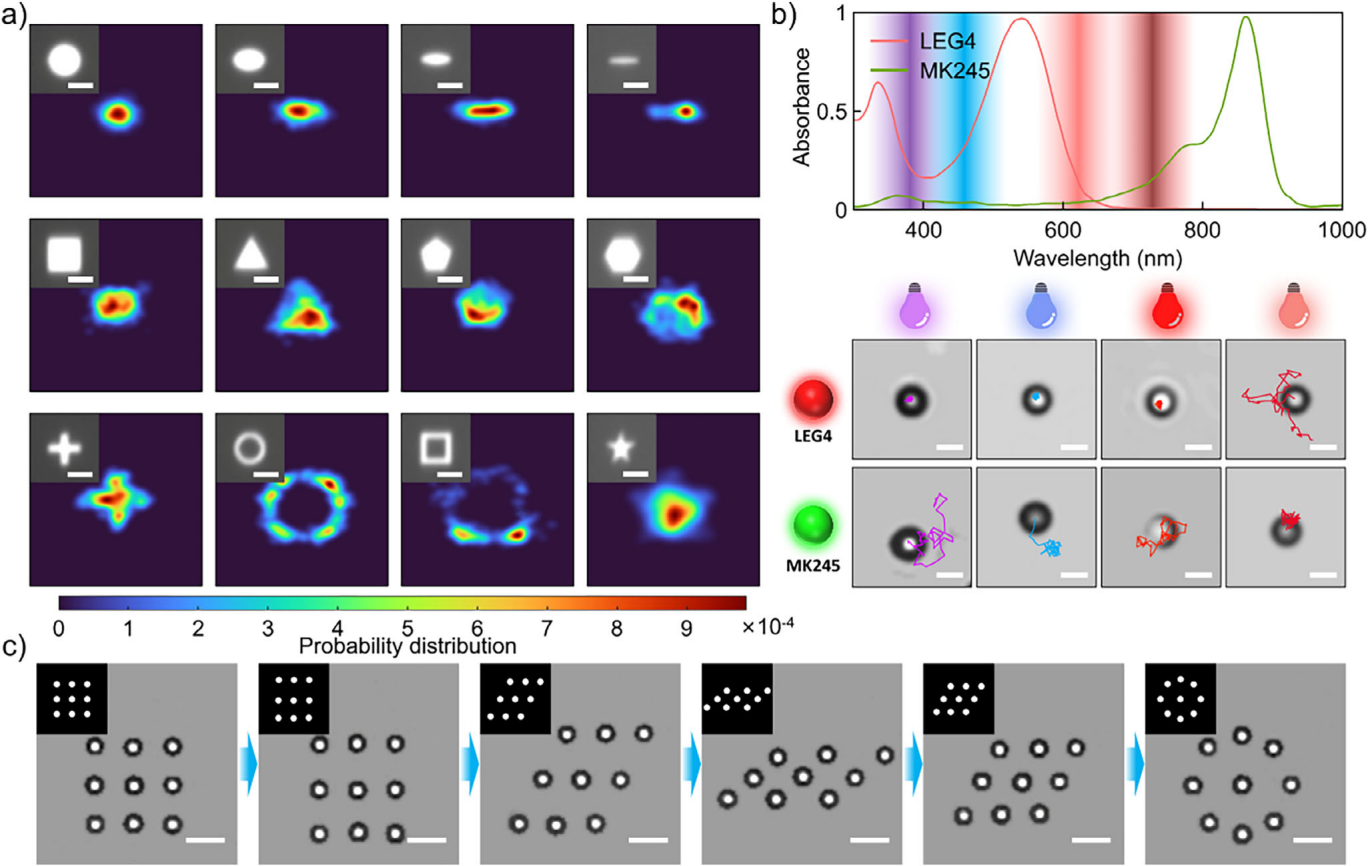
Versatile trapping and manipulation of LEG4‐TiO_2_ particles. (a) Statistical distribution of a single LEG4‐TiO_2_ particle confined in optical traps of defined shapes. (b) Spectral selectivity of the self‐trapping mechanism. Top panel: UV–vis absorption spectra of the LEG4 and MK245 sensitizer dyes. Bottom panels: Representative trajectories of LEG4‐TiO_2_ and MK245‐TiO_2_ particles within a circular light spot at selected wavelengths (385, 465, 625, and 730 nm), demonstrating wavelength‐dependent motility. (c) Reconfigurable assembly of colloidal lattices. Time‐lapse micrographs show nine particles being simultaneously arranged into distinct 2D lattices: square, rectangular, oblique, centered‐rectangular, hexagonal, and a custom centered‐circular cluster. Scale bars: (a,b) 2 µm, (c) 5 µm.

In contrast to conventional optical tweezers, which have limited beam‐shaping flexibility, our photoactive tweezers system offers exceptional spatial control. Using an LED‐based structured illumination system, we precisely define the projected pattern to arbitrarily confine particle motion. To demonstrate this, 12 distinct light patterns (each approximately 2 µm in size) were projected onto a single LEG4‐TiO_2_ particle. The resulting probability distribution, derived from particle trajectories, shows that the particle is effectively confined within a variety of shapes, including circles and ellipses (first row in Figure 4a), polygons (second row in Figure 4a), and more complex geometries such as “cross”, “ring” and “star” patterns (third row in Figure 4a), with representative trajectories available in Movie S4. The results indicate that the particle's trajectory can be effectively confined within the illuminated region, closely following the predefined light patterns.

The absorption spectrum of photoactive particles can also be tailored by functionalizing them with specific dye sensitizers, enabling wavelength‐selective tweezing [27, 36]. Here, we sensitized TiO_2_ particles with two different dyes: LEG4, which absorbs light from UV to ∼650 nm, and MK245, which absorbs in the near‐infrared region above ∼700 nm (Figure 4b, top panel). We compared the trapping behavior of LEG4‐ and MK245‐sensitized particles under a circular light spot of constant intensity at four wavelengths: 385, 465, 625, and 730 nm. Trajectory analysis (Figure 4b, lower panels) confirms that LEG4‐TiO_2_ is efficiently trapped at 385, 465, and 625 nm, but not at 730 nm. Conversely, MK245‐TiO_2_ shows a significant trapping effect only at 730 nm, with no effective confinement observed at the shorter wavelengths. These results demonstrate that trapping selectivity is dictated by the dye's absorption profile, allowing precise control by choosing the appropriate sensitizer. This wavelength selectivity opens avenues for applications such as particle sorting or selective cluster manipulation within mixed populations, further highlighting the versatility of this active tweezing technique (Movie S5).

Beyond trapping individual particles, our approach enables simultaneous manipulation of multiple active units. This allows us to assemble dynamic colloidal structures by programming the optical traps. We dynamically reconfigured an array of nine light spots into various lattice symmetries, including square, rectangular, oblique, centered‐rectangular, hexagonal, and centered‐circular configurations. As shown in Figure 4c and Movie S6, the nine LEG4‐TiO_2_ particles smoothly transition between predefined lattices, following the light pattern in real time. Additionally, the ability to assemble even larger quantities of particles is also demonstrated (Figure S4). The ability to reconfigure active particle assemblies provides a powerful tool for studying collective behaviors, phase transitions, and nonequilibrium dynamics in active matter systems [37, 38, 39, 40, 41].

### 2.4 Photoactive Tweezers for Cargo Transport and Micromachines

Building upon the fundamental trapping behavior, we advance the functionality of our photoactive tweezers system for manipulating cargo and micromachines, in which the confined active particle can function as a “tweezer arm” to capture, transport, or assemble other passive objects. Unlike conventional optical tweezers that use a highly focused laser beam to directly trap passive objects, our approach utilizes the phototactic self‐trapping of active particles as the primary manipulation mechanism. In this paradigm, multiple active particles can be simultaneously controlled by structured light spots, with each particle functioning as an independent and maneuverable unit. Through their collective and coordinated actions, these units enable the manipulation of objects significantly larger than individual particles, offering a unique and efficient strategy for micromanipulation and micro‐assembly under low optical intensities.

**FIGURE 5:**
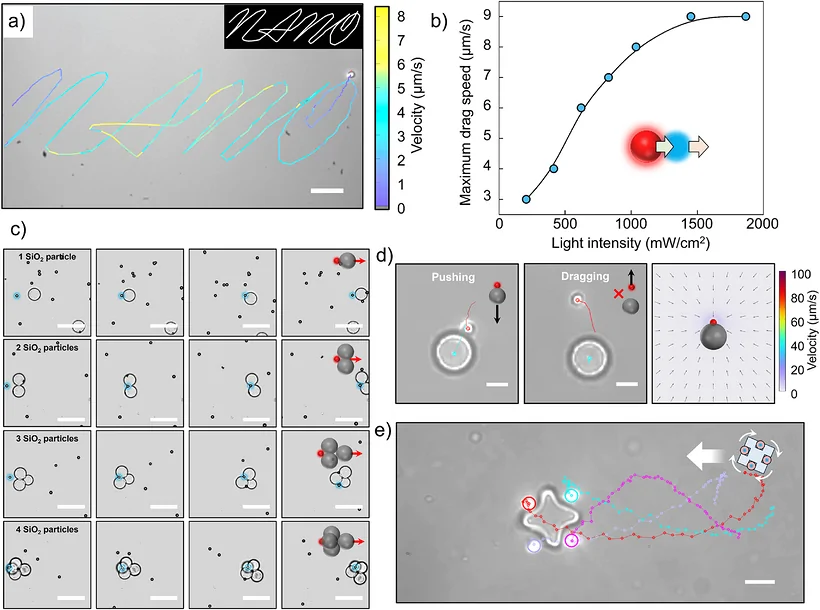
Programmable cargo transport and micromachines with photoactive tweezers. (a) Directed motion of a single TiO_2_ particle tracing a pre‐defined trajectory to spell “NANO”. (b) Measured maximum transport speed of a guided particle as a function of optical power intensity. The solid line represents a guide to the eye. In the inset model, blue circle represents a light spot, the red sphere represents an active particle, and the arrows indicate the movement of the light spot and the particle following it. (c) Transport of passive silica cargoes (10 µm diameter) comprising one to four particles, showcasing the ability to move objects significantly larger than the active carrier. The active particles are highlighted with blue circles. (d) Comparative trajectories of pushing (left panel) and dragging (middle panel) attempts, and the COMSOL simulation of the flow field (right) between the LEG4‐TiO_2_ particle and the silica cargo. The active particle and passive cargo and their trajectories are highlighted in red and blue, respectively. (e) Coordinated motion of a “micromachine” propelled by multiple active particles. The four active particles and their trajectories are highlighted in red, cyan, magenta, and light purple. Scale bars: (a) 10 µm, (c) 20 µm, (e) 5 µm.

We first demonstrate the precision and programmability of a single manipulation unit by directing it to trace the letters “NANO” along a complex, predefined path (Figure 5a and Movie S7). This requires real‐time control of the light spot's position to guide the phototactic particle. To ensure robust operation and avoid the particle escaping from the optical trap during such dynamic manipulation, it is crucial to characterize the maximum speed at which the guiding light spot can be moved. We therefore investigated the correlation between the dragging speed and the applied light intensity. As shown in Figure 5b, the maximum stable dragging speed, defined as the speed beyond which the particle can no longer follow the moving light spot, increases with light intensity from approximately 3 to 9 µm·s^−1^. This dependence is expected, as a higher light intensity generates a stronger phoretic velocity, enabling the particle to overcome viscous drag more effectively and track a faster‐moving trap. For the trajectory‐writing demonstration in Figure 5a, a light intensity of 1866 mW·cm^−2^ and a conservative maximum scanning speed of 8 µm·s^−1^ were selected to guarantee stability.

Not only can a single active particle undergo directed motion, but it can also act as a microscopic cargo transporter. As shown in Figure 5c and Movie S8, we successfully demonstrated that an active particle with a diameter of 2.5 µm can transport one, two, three, and even four such large cargo particles (10 µm in diameter). Our experiments reveal that successful transport is achieved exclusively through a direct pushing mechanism, in which the active particle makes physical contact from behind the cargo. In contrast, attempts to drag the cargo via long‐range hydrodynamic interaction, with the active particle positioned ahead, resulted in negligible displacement of the cargo, as evidenced by the comparative trajectories in Figure 5d (left and middle panels). This indicates that hydrodynamic entrainment alone is insufficient for sustained cargo transport, and the same conclusion holds for configurations involving two, three, or more cargo particles. The flow field around the active particle, obtained from COMSOL simulations (Figure 5d, right panel), is long‐ranged. However, its role in cargo transport appears to be secondary, while the dominant propulsive force arises from direct contact. A simple force balance analysis supports this: the photoactive trap stiffness (∼ 0.25 pN·µm^−1^) enables the active particle to generate a sufficient pushing force (∼ 0.1 pN) to overcome the viscous drag on a 10 µm cargo at speeds of ∼ 2 µm·s^−1^, requiring only a sub‐micrometer displacement within the trap.

Finally, to demonstrate multi‐unit cooperation, we constructed an integrated micromachine. A jigsaw‐puzzle‐shaped microstructure with four cavities was fabricated via photolithography (Figure S5). By embedding one active particle into each cavity, a multi‐propeller system was formed. Upon uniform illumination, the coordinated actuation of the four particles generates a net force. Through a precisely controlled rotational sequence of the light pattern, the micromachine is successfully propelled to move forward (Figure 5e and Movie S9). This experiment demonstrates the potential for executing complex tasks using collaborative “photoactive tweezers”, such as the targeted delivery of a microscopic payload. In this paradigm, the cargo is not directly “optically trapped”. Instead, it is mechanically through direct contact by the actively trapped particle, which functions as a mobile actuator. The platform therefore provides a versatile and powerful strategy for advanced micromanipulation, cargo transport, and the development of functional micromachines.

## 3 Conclusion

In conclusion, we have developed a versatile micromanipulation platform termed “photoactive tweezers” based on photoactive LEG4‐sensitized TiO_2_ particles and structured light illumination. When exposed to a light spot, these particles display positive phototaxis and become trapped at the beam center due to a self‐induced electrophoretic flow field. The effective trap stiffness, governed by the interplay between the particle diameter and the beam waist, follows a characteristic scaling law, which we explain using a self‐electrophoretic model and corroborate with COMSOL simulations. The flexibility of our approach is highlighted by its compatibility with arbitrarily shaped light patterns, wavelength‐selective operation using different dye sensitizers, and dynamic control of multiple particles in reconfigurable lattices. Furthermore, by employing these active particles as independent manipulation units, we demonstrate complex functionalities, including guided motion along predefined paths, transport of cargo exceeding the particle's own size, and coordinated action in an assembled micromachine. This “photoactive tweezers” strategy leverages low optical intensity for indirect manipulation, demonstrating a paradigm shift from directly manipulating targets with light to programming active matter systems with light to perform manipulation. This “active matter as a tool” approach could enable new capabilities in biocompatible handling, parallel manipulation, and the operation of synthetic micromachines.

## 4 Experimental Section

*Chemicals and materials*: Materials include LEG4 dye sensitizer (3‐{6‐{4‐[bis(2′,4′‐dibutyloxybiphenyl‐4‐yl)amino‐]phenyl}‐4,4‐dihexyl‐cyclopenta‐[2,1‐b:3,4‐b′]dithiophene‐2‐yl}‐2‐cyanoacrylic acid, product code DN‐F05, Dyenamo AB, Stockholm, Sweden), MK245 dye sensitizer (3‐(2‐((*E*)‐2‐((*E*)‐3‐((*Z*)‐2‐(3‐(2‐carboxyethyl)‐1,1‐dimethyl‐1,3‐dihydro‐2*H*‐benzo[e]indol‐2‐ylidene)ethyl‐idene)‐2‐chlorocyclohex‐1‐en‐1‐yl)vinyl)‐1,1‐dimethyl‐1*H*‐benzo[e]indol‐3‐ium‐3‐yl)propanoate, product code DN‐FI07, Dyenamo AB, Stockholm, Sweden) for sensitization, ferrocene (1 mol/L in acetonitrile) as a redox shuttle, titanium(IV) isopropoxide (AR grade) and acetone (AR grade) for TiO_2_ synthesis.

*LEG4‐TiO_2_ microparticle suspensions*: The 2.5 µm TiO_2_ microspheres are synthesized by a solvothermal method, followed by a hydrothermal pre‐crystallization process and an annealing process at 450°C. The particles are then sensitized with a saturated solution of the dye sensitizers. The preparation details can be found in the Supporting Information. The as‐prepared samples are washed three times with acetonitrile and redispersed in 0.1 M solution of ferrocene in acetonitrile for use.

*Structured light control and particle tracking*: The control of structured light patterns is realized by a computer package designed for the Four‐Channel Digital Light Processing Projection System, which reads and projects images from the computer's split screen (Figure S6). A MATLAB code is developed to realize the real‐time projection and manipulation of the projected patterns by generating images on the split screen (Movie S10). The experiments are conducted on an Olympus IX81 inverted microscope with a 100× oil immersion objective, where the images and videos are recorded by a BASLER a2A1920‐160umBAS CCD camera. The projection system is attached to the epifluorescence port on the microscope. The particle tracking velocimetry, KDE calculation of probability distribution, potential calculation, and fitting are conducted in MATLAB. All codes are available upon request.

### 4.1 Data Analysis and Error Quantification

#### 4.1.1 Mean Square Displacement (MSD)

The trajectories of individual particles were obtained using particle tracking velocimetry. The time‐averaged MSD was calculated as MSD(τ) = 〈|*r*(*t* + τ) − *r*(*t*)|^2^〉, where *r*(*t*) is the particle's position at time *t*, and τ is the time lag. For freely diffusing particles (no illumination), the MSD was fitted to the equation to extract the diffusion coefficient *D*_0_.

#### 4.1.2 Trapping Potential and Stiffness Analysis

The spatial probability distribution of a trapped particle was calculated from its trajectory using Kernel Density Estimation (KDE). The effective potential was derived via Boltzmann inversion *U_e_* (*x*,*y*) =   − *k_B_T*ln (*f*(*x*,*y*)). The coordinates were then converted to radial distance *r* from the trap center. The radial potential *U_e_* (*r*) was fitted to a harmonic form $U_{e}\left(r\right)=\frac{1}{2}k_{e}r^{2}$ using a nonlinear least‐squares fit algorithm via Origin's built‐in fitting function. The error bars for the effective trap stiffness in Figure 2c,d represent the standard error of the fit.

## Author Contributions

**Shiwei Zhang**: validation, methodology. **Jingyuan Chen**: conceptualization, investigation, writing – original draft, data curation. **Jinyao Tang**: conceptualization, funding acquisition, validation, supervision, resources, project administration, writing – review and editing. **Changjin Wu**: methodology. **Binglin Zeng**: software, methodology, conceptualization, writing – original draft. **Donghao Cui**: methodology. **Kai Lou**: software, data curation, resources. **Pei Xu**: methodology. **Wei Wang**: conceptualization, supervision. **Qingxin Guo**: formal analysis, visualization.

## Conflicts of Interest

The authors declare no conflicts of interest.

## Supporting information




**Supporting File 1**: adma74255‐sup‐0001‐SuppMat.docx.

**Supporting File 2**: adma74255‐sup‐0002‐MovieS1‐S10.zip.

## Data Availability

The data that support the findings of this study are available from the corresponding author upon reasonable request.
