# Supplementary material for: Photoactive Tweezers
Source: Adv Mater. 2026 Jul 23;38(49):e74255. doi: 10.1002/adma.74255 (PMC13532508; doi:10.1002/adma.74255)
Supplement: Supplementary file 1 [file ADMA-38-e74255-s002.docx]

**Photoactive Tweezers**

Jingyuan Chen^1,2^, Qingxin Guo^1^, Shiwei Zhang^1^, Donghao Cui^1^, Changjin Wu^1^, Pei Xu^1^, Wei Wang^3^, Kai Lou^4^, Binglin Zeng*^1,5^ and Jinyao Tang*^1,2,5^

^1^Department of Chemistry, The University of Hong Kong, Pokfulam, Hong Kong, China

^2^Materials Innovation Institute for Life Sciences and Energy (MILES), HKU-SIRI, Shenzhen, China

^3^Department of Materials Science and Engineering, Harbin Institute of Technology, Shenzhen, Guangdong, China

^4^Shenzhen Kayja-Optics Technology Co. Ltd., Shenzhen, Guangdong, China

^5^State Key Laboratory of Synthetic Chemistry, The University of Hong Kong, Kowloon, Hong Kong, China

**Supplementary Methods and Discussions**

1. Preparation of LEG4-TiO_2_ microparticles

2. COMSOL simulation of self-electrophoresis

3. Derivation of the power scaling of trap stiffness

4. Assembly of large-scale crystal lattice

5. Preparation of micropattern with photolithography

6. Digital light projection control system

**Supplementary Figures**

Figure S1. Characterization of the synthesized TiO_2_

Figure S2. COMSOL simulation results

Figure S3. Modelling of the phototactic self-trapping effect

Figure S4. Large-scale arbitrary crystal lattice assembly

Figure S5. Fabrication of jigsaw-puzzle-shaped microstructure

Figure S6. Digital light projection control system

**Movies**

**Movie S1:** Self-trapping effect of 2.5 μm LEG4-TiO_2_ particle

**Movie S2:** Self-trapping effect of LEG4-TiO_2_ particle under different particle sizes and optical trap sizes

**Movie S3:** Self-trapping effect of 2.5 μm LEG4-TiO_2_ particle under increasing light intensity

**Movie S4:** Self-trapping effect of LEG4-TiO_2_ particles under different light spot shapes

**Movie S5:** Self-trapping effect of LEG4-TiO_2_ and MK245-TiO_2_ under different wavelengths of light

**Movie S6:** Crystal transformation of a 3×3 LEG4-TiO_2_ particle array

**Movie S7:** Control of a LEG4-TiO_2_ particle writing “NANO”

**Movie S8:** Manipulation of large cargoes with LEG4-TiO_2_ particle

**Movie S9:** Collective maneuvering of a micromachine with 4 LEG4-TiO_2_ particles
**Movie S10:** Manual control demonstration of the photoactive tweezers system

**Supplementary Methods and Discussions**

**1. Preparation of LEG4-TiO_2_ Microparticles**

The 2.5 μm TiO_2_ microspheres are synthesized by a solvothermal method. Frist, 2.54 mL of titanium isopropoxide (TTIP) is added into acetone with a ratio of 2.5:30 and a total volume of 33 mL, the solution is then placed in ultra sonification for 10 minutes, resulting in a pale, semi-transparent mixture. The mixture is then transferred into a 50 mL Teflon lined autoclave, which is heated in an oven for 12 h under 200 ℃. The resulting yellowish precipitates is washed with isopropanol for 3 times by resuspending and centrifuging. The same procedure is repeated in water for another 3 times. To perform further purification and pre-crystallization to maintain the porous structure, the finial aqueous suspension with a total volume of 33 mL is transferred to a Teflon lined autoclave and heated to 160 ℃ for 24 h. The obtained white precipitate is further dried in 100 ℃ oven overnight and then annealed under 450 ℃ for 1 h in a furnace. **Fig. S1a-b** shows the SEM and TEM images.

The LEG4-TiO_2_ particles are prepared by loading porous TiO_2_ powder with the dye sensitizer LEG4. A saturated LEG4 solution with magenta color is prepared with isopropanol (IPA). 1 mg of the porous TiO_2_ powder is directly dispersed in 1 mL of the saturated LEG4 solution and then allowed to stand overnight. The suspension is then centrifuged and washed with IPA for three times, resulting in a reddish particle (**Fig. S1c**).

**Figure S1.** **Characterization of the synthesized TiO_2_. a,** SEM image of solvothermally synthesized TiO_2_ particles with an averaged diameter of 2.5 μm. The inset shows the high-resolution image of a single particle. **b,** TEM image revealing the porous internal structure of the TiO_2_ particle formed after hydrothermal pre-crystallization and subsequent annealing. **c,** Optical microscopic image of the LEG4-dyed TiO_2_ particles. Scale bars: **(a)** 10 μm (inset 1 μm), **(b)** 200 nm, **(c)** 10 μm.

**2. COMSOL Simulation of Self-Electrophoresis**

The phototactic behavior of LEG4-TiO_2_ is simulated via COMSOL Multiphysics package. In brief, a simplified 2D model is constructed to simulate the self-electrophoresis under light spot (**Fig. S2a**). To establish the model, three modules are incorporated: diffusion of dilute species, electrostatic and creeping flow. When the LEG4-TiO_2_ particle is placed under Gaussian light spot, the illuminated part generates holes that oxidizes ferrocene (Fe(Cp)_2_) into ferrocenium (Fe(Cp)_2_^+^), while the electrons in the shaded part reduces Fe(Cp)_2_^+^ into Fe(Cp)_2_, generating a net ion flow. The diffusion of Fe(Cp)_2_^+^ created is numerically calculated by the first module, where the electric field caused by the diffusion of the Fe(Cp)_2_^+^ is modelled by the second module. Finally, the tangential component of the electric field will drive the ions in the electrical double layer on the particle surface and produce electroosmotic flow, which is simulated by the third module.

For the calculation of transport of dilute species, the distribution of H^+^ and OH^-^ can be affected by diffusion, convection and electrophoresis under the electric field, which can be resolved by the continuity equation at steady state,

$$\nabla\cdot\boldsymbol{J}=\boldsymbol{u}\nabla c-D\nabla^{2}c-\frac{zFD\nabla\cdot(c\nabla\varphi)}{RT}$$

where ***J*** is the flux of Fe(Cp)_2_^+^ ion, ***u*** is the fluidic velocity, *F* is the Faraday constant, *φ* is the electrostatic potential, *R* is the gas constant, *T* is the temperature, *c*, *D* and *z* are the concentration, diffusion coefficient and the charge of Fe(Cp)_2_^+^, respectively. The diffusion coefficient of Fe(Cp)_2_^+^ and photocurrent can be found in literature^1-3^. To simulate the effect of the Gaussian light beam, since the reaction flux scales linearly with the light intensity with $j\left( r \right)=\beta I(r)$, the reaction flux on the illuminated hemisphere also follows a Gaussian profile, which is defined by a Gaussian prefactor $G=exp(-\frac{2\left( x+dx \right)}{w_{0}^{2}})$. The Gaussian light beam for simulation and the simulated concentration distribution of Fe(Cp)_2_^+^ are shown in **Fig. S2b** and **c_._**

The electrostatic potential *φ* ($\boldsymbol{E}=-\nabla\varphi$) from Eq. (1) can be calculated from the Poisson equation,

$${-\varepsilon}_{0}\varepsilon_{r}\nabla^{2}\varphi=\rho_{e}=FZc Eq. (2)$$

where *ε_0_* is the vacuum permittivity and *ε_r_* is the relative permittivity of acetonitrile. *Z=1* for Fe(Cp)_2_^+^. *ρ_e_* is the volumetric charge density, *F* is the Faraday constant, *c* is the concentration of Fe(Cp)_2_^+^. The resulting electric field distribution are plotted in **Fig. S2d_._**

In low Reynolds number system, the fluid flow outside the electric double layer is governed by Stokes equation at steady state and the continuity equation for the incompressible fluid,

$$-\nabla p+\mu\nabla^{2}\boldsymbol{u}=0$$

$$\nabla\cdot\boldsymbol{u}=0$$

where *μ*, ***u*** and *p* are the dynamic viscosity of acetonitrile, velocity and pressure, respectively. The initial value of ***u*** and *p* are zero in our simulation.

In the fluid flow module, the boundary condition of the particle surface is set to be the electroosmotic boundary which is dominated by the tangential component of the electric field by

$$\boldsymbol{E}_{\boldsymbol{t}}=\boldsymbol{E}-(\boldsymbol{E}-\boldsymbol{n})\cdot\boldsymbol{n}$$

where ***E_t_*** is the tangential component of the electric field strength ***E***. The electroosmotic velocity is then governed by

$$\boldsymbol{u}=-\frac{\varepsilon_{r}\varepsilon_{0}\zeta}{\mu}\boldsymbol{E}_{\boldsymbol{t}}$$

*ζ* here represents the zeta potential of the particle, which is set to be -20 mV for LEG4-TiO_2_ particle.

To extract the trap stiffness from the simulation, the hydrodynamic pressure exerted on the particle is first converted into force by Stokes’ law $F=6\pi r\eta v$, and the position of the light spot center x is scanned to extract the restoring force $F(x)$. The trap stiffness $k$ is then extracted by linear fitting $F\left( x \right)=kx$.

**Figure S2. COMSOL simulation results. a,** Photo-electrochemical reaction occurring at the LEG4-TiO_2_ particle interface. **b,** Gaussian profile of the incident light beam used in the simulation. **c,** Simulated concentration distribution of Fe(CP)_2_^+^ around the particle. **d,** Electric field magnitude surrounding the particle.

**3. Derivation of the Power Scaling of Trap Stiffness**

Consider a spherical photoactive particle with a diameter $d$ in a non-uniform light field. The surface reaction generates a local ion flux can be regarded as being proportional to the incident light intensity^3^:

$$j=\beta I$$

where $\beta$ is a photochemical reaction rate constant.

We assume that the particle is small enough, the light intensity on the particle surface can thereby be expended as:

$$I\left( \theta\right)\approx I_{0}+\frac{1}{2}d\nabla I\cos\theta$$

where $I_{0}$ is the light intensity at the beam center, $\theta$ is the polar angle on the particle. Therefore, the ion flux can be written as:

$$j\left( \theta\right)={\beta I}_{0}+\frac{1}{2}\beta d\nabla I\cos\theta$$

The non-uniform portion of the flux (proportional to $\cos\theta$) drives particle motion, while the uniform portion (${\beta I}_{0}$) produces no net gradient. Under steady-state conditions ($\nabla^{2}c=0$), the ion concentration field satisfies the diffusion equation with the following boundary conditions:

$$-D\left. \frac{\partial c}{\partial r} \right|_{r=\frac{d}{2}}=j\left( \theta\right)$$

where D is diffusion coefficient of the ion. By only considering the non-uniform portion and solving the equation, we can get the surface concentration profile:

$$c\left( a,\theta\right)=c_{\infty}+\frac{\beta d\nabla I}{4D}\frac{d}{2}\cos\theta=c_{\infty}+\frac{\beta d^{2}\nabla I}{8D}\cos\theta$$

where $c_{\infty}$ is the concentration at infinity. The surface tangential concentration gradient is:

$$\nabla_{s}c=\frac{2}{d}\frac{\partial c}{\partial\theta}\boldsymbol{e}_{\theta}=-\frac{\beta d\nabla I}{4D}\sin\theta\boldsymbol{e}_{\theta}$$

The concentration gradient generates an electric field $E$. Under the assumption of electrical neutrality, the electric field is related to the concentration gradient by:

$$E=-\frac{k_{B}T}{e}\frac{\nabla c}{c_{0}}$$

where $k_{B}$ is the Boltzmann constant, $T$ is temperature, $e$ is the elementary charge, $c_{0}$ is the averaged ion concentration (roughly equals to $c_{\infty}$). Since the tangential component of electric field $E_{t}$ is proportional to the surface tangential concentration gradient $\nabla_{s}c$, we have

$$E_{t}=-\frac{k_{B}T}{e}\frac{\nabla_{s}c}{c_{0}}=\frac{k_{B}T}{e}\frac{\beta d\nabla I}{4Dc_{0}}\sin\theta\boldsymbol{e}_{\theta}$$

The electrophoresis slip velocity is:

$$v_{s}=\frac{\varepsilon\zeta}{\eta}E_{t}$$

where $\varepsilon$ is the dielectric constant, $\zeta$ is the zeta potential of the particle, $\eta$ is the fluid viscosity. The particle velocity $v_{p}$ is the integral of the sliding velocity over the surface:

$$v_{p}=\frac{1}{\pi d^{2}}\int v_{s}dS$$

Substituting $v_{s}$ and $E_{t}$ into the equation and integrating over the spherical surface, we have

$$v_{p}=\frac{\varepsilon\zeta}{\eta}\frac{k_{B}T}{e}\frac{\beta d}{4Dc_{0}}\nabla I\propto d\nabla I$$

where the particle diameter $d$ dependence of $v_{p}$ is experimentally measured, as plotted in **Fig. S3**. In the overdamped regime, the propulsion force is balanced by the Stokes drag force, which is then expressed by:

$$F_{drag}=3\pi\eta dv_{p}=3\pi\eta d\frac{\varepsilon\zeta}{\eta}\frac{k_{B}T}{e}\frac{\beta d}{4Dc_{0}}\nabla I=3\pi\varepsilon\zeta\frac{k_{B}T}{e}\frac{\beta d^{2}}{4Dc_{0}}\nabla I$$

For a Gaussian beam, the light intensity profile is

$$I(r)=I_{0}\exp( -\frac{2r^{2}}{w_{0}^{2}})$$

where $I_{0}=\frac{2P}{\pi w_{0}^{2}}$, *P* is the input power from the light source, $w_{0}$ is the beam waist. Near the beam center ($r\ll w_{0}$), the magnitude of light intensity gradient can be written as

$$\nabla I=\frac{2Pr}{\pi w_{0}^{4}}$$

Inserting into the expression of $F_{drag}$, we have

$$F_{drag}=3\pi\varepsilon\zeta\frac{k_{B}T}{e}\frac{\beta d^{2}}{4Dc_{0}}\nabla I=3\pi\varepsilon\zeta\frac{k_{B}T}{e}\frac{\beta d^{2}}{4Dc_{0}}\frac{2Pr}{\pi w_{0}^{4}}=3\varepsilon\zeta\frac{k_{B}T}{e}\frac{\beta d^{2}}{2Dc_{0}}\frac{Pr}{\pi w_{0}^{4}}$$

The trap stiffness $k$ is therefore derived as:

$$k=\frac{dF_{drag}}{dr}=\frac{3\beta P\varepsilon\zeta k_{B}T}{2\pi eDc_{0}}\frac{d^{2}}{w_{0}^{4}}\propto\frac{d^{2}}{w_{0}^{4}}$$

which yields the experimentally observed scaling law $k\sim d^{2}/w_{0}^{4}$.

**Figure S3. Modelling of the phototactic self-trapping effect. a,** Configuration of the model. **b,** Experimentally measured phototactic speed of particles as a function of particle size.

**4.** **Assembly of Large-Scale Crystal Lattice**

We explored the assembly of larger colloidal lattices with the “active optical tweezers”. The five Bravais lattices with 20 particles in total are first assembled, along with a special lattice representing a typical 2D material lattice of graphene. This approach might provide a powerful tool to further perform theoretical investigation of 2D materials (**Fig. S4a**). The assembly of large square lattice with 144 particles is easily realized and can be further controlled individually, demonstrating a simple yet powerful technology without the need of light beam splitting like optical tweezers, which will limit the maximum number of manipulated particles (**Fig. S4b**).

**Figure S4. Large-scale arbitrary crystal lattice assembly. a,** Formation of square, centered-rectangular, hexagonal, rectangular, oblique, and graphene lattices by assembling 20 particles.. **b,** Assembly of a large 12×12 square lattice comprising 144 particles. Scale bars: 5 μm for both (a) and (b).

**5. Preparation of Micropattern with Photolithography**

To prepare the micropatterns, silicon wafers with a 300 nm silicon dioxide layer are first cut into 30 mm squares, which are sequentially ultrasonically cleaned with acetone, IPA, and DI water for 15 minutes each, followed by surface drying using a nitrogen gun. Subsequent surface cleaning is performed with O₂ plasma for 3 minutes. A layer of PMMA A4 electron beam resist is first spin-coated as the liftoff layer at a rotation speed of 4000 rpm for 45 seconds, and then baked at 180 °C for 90 seconds. Thereafter, a mixture of cyclopentanone and SU-8 3005 (1:1 v/v) is spin-coated to achieve a thinner SU-8 thickness; the rotation speed is set to 4000 rpm for 30 seconds. This is followed by baking at 65 °C for 1 minute and subsequent baking at 95 °C for 2 minutes. Subsequently, the pattern depicted in **Fig. S5a** is patterned using the SMART PRINT UV Maskless Lithography System. A 10× objective lens is employed for lithography, with a single exposure time of 0.32 seconds and the light intensity set to 50%. After lithography, a post-exposure bake is conducted at 95 °C for 3 minutes. Propylene glycol methyl ether acetate (PGMEA) is used as the developer, **Fig. S5b** shows the prepared patterns on the substrate. The micropatterns are lifted off by washing with acetone, and the resultant product is collected by rapid centrifugation, followed by washing with acetonitrile three times, yielding an acetonitrile dispersion containing the target-shaped structures.

**Figure S5. Fabrication of jigsaw-puzzle-shaped microstructure. a,** The single lithography pattern and its dimensions. **b,** Microscopic image of the prepared substrates with micropatterns. Inset shows the image of the wafer with large quantity of micropatterns. Scale bars: **(b)** 50 μm.

**6. Digital Light Projection Control System**

The control system for our active optical tweezers is based on an inverted microscope (Olympus IX81), four LED light sources with their corresponding DMD modules (integrated within a Four-Channel DLP Projection System, Kayjia-Optics), and a software-based control process running on a computer. Observation was performed using a BASLER a2A1920-160umBAS CCD camera (100 fps max) via a 100× oil-immersion objective (NA=1.35). The core concept is to use computer-generated digital images to program and control the light field projected onto the sample in real-time, thereby achieving optical manipulation. The entire control process is implemented through the interaction between a MATLAB control program and the manufacturer-provided digital light control program on the computer, which interfaces with the hardware. **Fig. S6a** illustrates the system connections. The control procedure is as follows.

First, the digital pattern generation. A custom MATLAB program dynamically generates grayscale images based on experimental requirements, such as pre-defined motion sequences, real-time mouse commands, dynamic videos, or static arrays. The shape and position information in these images directly define the geometry, intensity, and spatial distribution of the optical traps.

Next, the pattern allocation and display. The generated images are sent by the MATLAB program to four designated “system screens” of the computer’s extended desktop. Each screen is assigned to a corresponding channel of the DLP projector. The manufacturer’s digital light control program captures the content of these screens and relays the signals to control the hardware, including the input light power and the projected image.

Finally, the optical projection and calibration. The DLP system projects the optical pattern. The modulated light from the four channels is introduced into the microscope’s epifluorescence port and focused onto the sample plane by the objective, converting the digital pattern into a structured light field. A critical calibration step links digital pixels to physical space: a grid pattern with known pixel spacing is projected and imaged onto a microscope graticule. By measuring the physical distance corresponding to the projected pixels, the calibration coefficient (µm/pixel) for the objective is determined, enabling precise positional control. The corresponding control flow is shown in **Fig. S6b**.

**Figure S6. Digital light projection control system. a,** Configuration of the setup and connection. **b,** Control loop of the digital light projection system.

**References**

1	Wang, Y., Rogers, E. I. & Compton, R. G. The Measurement of the Diffusion Coefficients of Ferrocene and Ferrocenium and Their Temperature Dependence in Acetonitrile Using Double Potential Dtep Microdisk Electrode Chronoamperometry. *J. Electroanal. Chem.* **648**(1), 15-19 (2010).

2	Zheng, J. *et al.* Orthogonal Navigation of Multiple Visible-Light-Driven Artificial Microswimmers. *Nat. Commun*. **8**(1), 1438 (2017).

3	Wang, J. *et al.* Rational Design of Reversible Redox Shuttle for Highly Efficient Light-Driven Microswimmer. *ACS nano* **14**(3), 3272-3280 (2020).
